# Anterior Spinal Artery Syndrome: Reversible Paraplegia after Minimally Invasive Spine Surgery

**DOI:** 10.1155/2014/205732

**Published:** 2014-08-20

**Authors:** J. Bredow, J. Oppermann, K. Keller, F. Beyer, C. K. Boese, K. Zarghooni, R. Sobottke, P. Eysel, J. Siewe

**Affiliations:** ^1^Department of Orthopedics and Trauma Surgery, ZKS (BMBF 01KN1106), University Hospital of Cologne, Kerpener Straße 62, 50924 Cologne, Germany; ^2^Department of Orthopedics, Medical Center City Aachen GmbH, 52146 Würselen, Germany

## Abstract

*Background Context*. Percutaneous balloon kyphoplasty is an established minimally invasive technique to treat painful vertebral compression fractures, especially in the context of osteoporosis with a minor complication rate. *Purpose*. To describe the heparin anticoagulation treatment of paraplegia following balloon kyphoplasty. *Study Design*. We report the first case of an anterior spinal artery syndrome with a postoperative reversible paraplegia following a minimally invasive spine surgery (balloon kyphoplasty) without cement leakage. *Methods*. A 75-year-old female patient underwent balloon kyphoplasty for a fresh fracture of the first vertebra. *Results*. Postoperatively, the patient developed an acute anterior spinal artery syndrome with motor paraplegia of the lower extremities as well as loss of pain and temperature sensation with retained proprioception and vibratory sensation. Complete recovery occurred six hours after bolus therapy with 15.000 IU low-molecular heparin. *Conclusion*. Spine surgeons should consider vascular complications in patients with incomplete spinal cord syndromes after balloon kyphoplasty, not only after more invasive spine surgery. High-dose low-molecular heparin might help to reperfuse the Adamkiewicz artery.

## 1. Introduction

Percutaneous balloon kyphoplasty is a minimally invasive technique to treat painful vertebral compression fractures, especially in the context of osteoporosis [1–4].

Under general or local anesthesia, a balloon is inserted through a needle into the vertebral body to create a cavity which is filled with acrylic cement, once the balloon is removed, to stabilize the vertebral body [5]. In this procedure, cement leakage is the most frequent complication, occurring in 7% to 9% of the cases. In about 1% to 5% of the complicated cases, cement leaks into the venous circulation, inducing pulmonary embolism [6, 7].

In a meta-analysis of complications after percutaneous treatment of vertebral compression fractures, Lee et al. reported a rate of 0.9% for kyphoplasty [4]. Here, most symptomatic cement leaks caused single level radiculopathy and were treated either with steroid injection or surgical decompression [4]. Severe neurologic deficits have also been described in this context [4, 8, 9].

The vascular supply of the spinal cord relies on three longitudinal arterial trunks: the anterior spinal artery, which originates at cervical levels from the vertebral arteries, and the posterolateral spinal arteries. At the thoracic and lumbar levels, the anterior spinal artery is additionally supplied by segmental aortic vessels [10, 11]. The most important feeding artery of the thoracolumbar spinal cord is the great anterior radiculomedullary artery, also known as the Adamkiewicz artery [12, 13]. This artery supplies the lower two-thirds of the spinal cord via the anterior spinal artery [14, 15]. If this artery is injured or unintentionally interrupted (dominant vascular supply to the anterior spinal cord), it might lead to ischemia of the ventral horn, ventral commissura, and the sympathetic centers of the intermediolateral region; it manifests as anterior spinal artery syndrome with impaired motor and sensory function of the bilateral lower extremities and loss of urinary and fecal continence [10, 12, 16, 17].

The anatomical location of this vessel is a matter of concern for surgeons since its ligation might significantly reduce the blood supply to the cord [18]. Spinal cord ischemia with paraplegia is rarely reported after segmental vessel ligation and if at all, after anterior thoracolumbar spinal surgery [18–21]. In the present literature on minimally invasive surgery, no anterior spinal artery syndrome has been yet described as complication.

## 2. Materials and Methods

**Figure 1:**
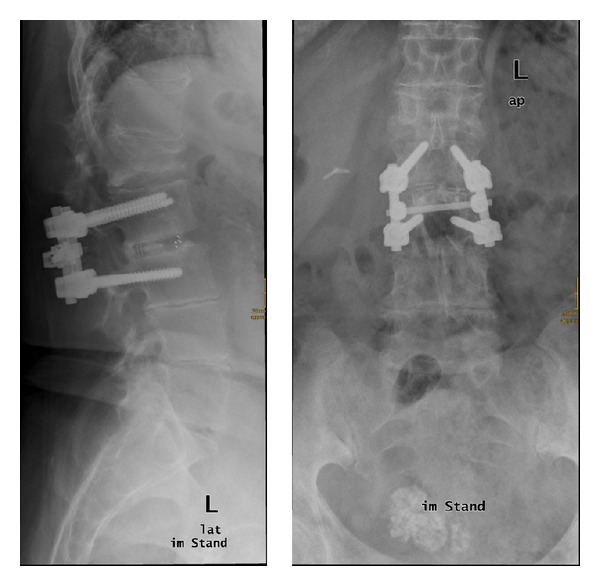
Preoperative X-ray in standing position in sagittal and antero-posterior plane and AP. Fresh fracture in L1. Prior operation was performed in L2/3 with a posterior lumbar intervertebral fusion (PLIF) 2 years ago.

We describe a 75-year-old female patient who presented with a fracture of the first vertebra (Type A1 Magerl) of the lumbar spine (L1) related to postmenopausal osteoporosis. A fresh fracture was diagnosed in the MRI above posterior lumbar intervertebral fusion (PLIF) in L2/3 which was operated 2 years earlier because of an erosive osteochondrosis (Figure 1). She also suffered from arterial hypertension, cardiac insufficiency (NYHA II), chronic atrial fibrillation, compensated renal insufficiency, adiposity, and insulin-dependent diabetes mellitus (Type II).

Bipedicular balloon kyphoplasty was performed using polymethylmethacrylate bone cement. On both sides, the balloons were inflated under visual and pressure control. 2 mL of cement per side, 4 mL in total, was inserted under fluoroscopic monitoring in the vertebra. Intraoperative fluoroscopy showed no evidence of cement leakage, indicating that the intervention had been well performed.

## 3. Results

**Figure 2:**
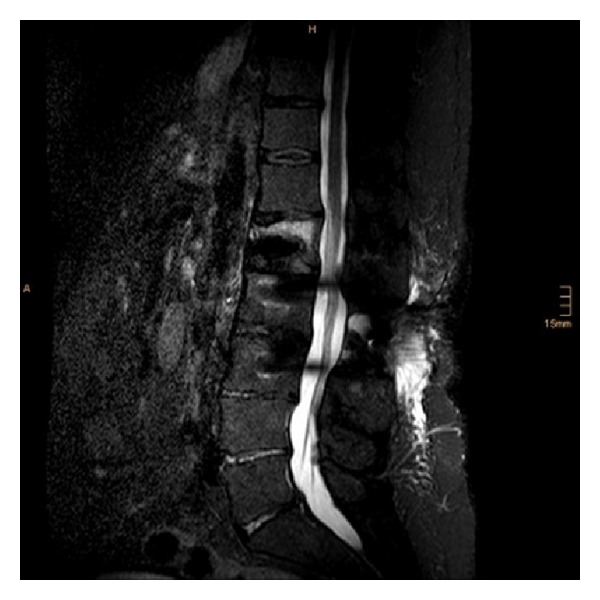
Postoperative sagittal MRI (STIR).

**Figure 3:**
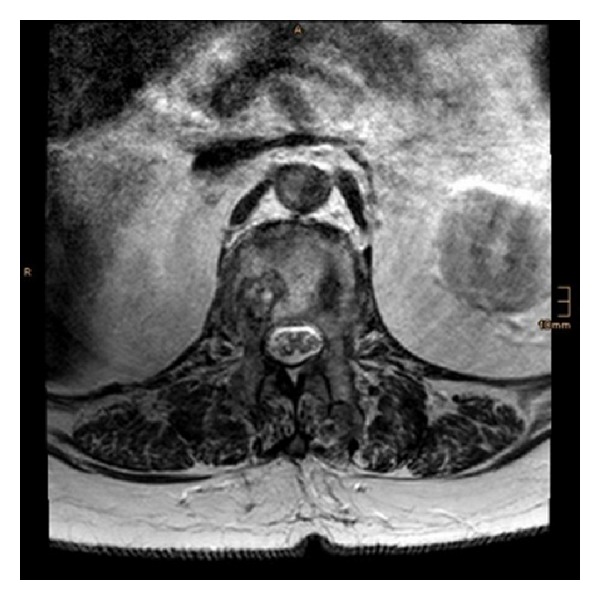
Postoperative axial MRI (T2-weighted) at the level of L1.

**Figure 4:**
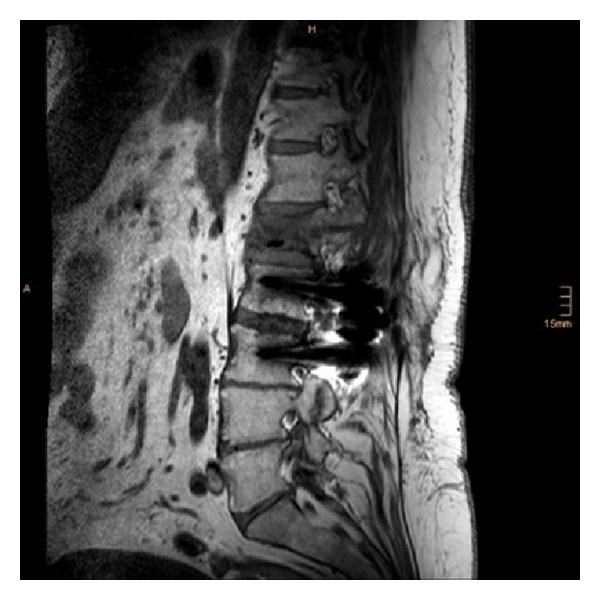
Postoperative sagittal MRI (T1-weighted) at the level of the pedicles (left).

**Figure 5:**
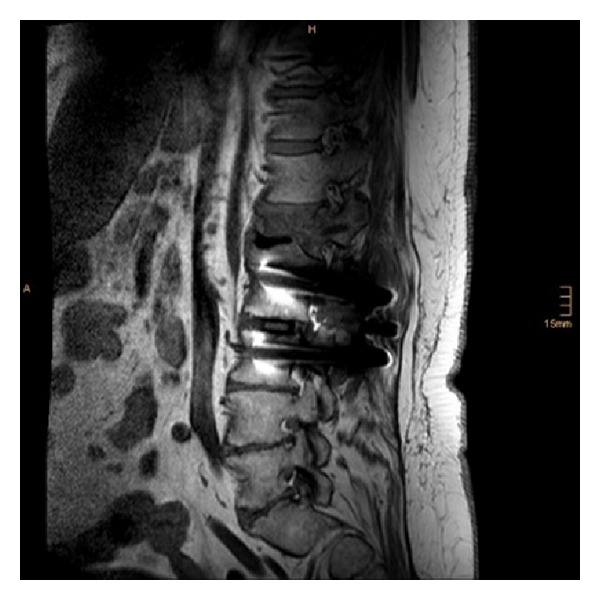
Postoperative sagittal MRI (T1-weighted) at the level of the pedicles (right).

Postoperatively the patient developed a motor paraplegia of the lower extremities and a loss of pain and temperature sensation with retained proprioception and vibratory sensation. The patient complained of belt-like pain; the tendon reflexes were without pathological findings on the arms, and patellar reflex was weakened; Achilles reflex expired. There was a moderate hypesthesia caudally from L1. A promptly carried-out MRI excluded causes such as cement in the spinal canal, intraspinal hematoma, incorrect transpedicular approach, intraspinal tightness, or myelopathy (Figures 2, 3, 4, and 5). The neurologic consultant diagnosed an anterior spinal artery syndrome and recommended treatment with 15.000 IU low-molecular heparin as intravenous bolus. Six hours later, the paraplegia had completely regressed. On the next day, the patient could be mobilized without restrictions.

## 4. Discussion

Paraplegia after (anterior) spine surgery remains rare. There are several major risk factors for postoperative neurologic deficit, for example, spinal deformity correction or hypotension during surgery [10, 19]. Paraplegia resulting from vessel ligation has been reported by several authors [10, 18–21]. Wadouh et al. found that ligation of all of the segmental arteries from L1 to S1 (7 levels), including the level of the Adamkiewicz artery, resulted in paraparesis in 3 pigs and paraplegia in 2 [22]. Using a dog model, Kato et al. demonstrated that interruption of bilateral segmental arteries at more than 4 consecutive levels, including the level of the Adamkiewicz artery, risks producing ischemic spinal cord dysfunction [23].

On the other hand, in other cases, the Adamkiewicz artery was sectioned at the nerve root without any neurologic consequences, and, in 4 cases of spinal tumors, a new feeding artery was found in the postoperative angiogram [12]. In 3 other cases, no postoperative neurologic deficit was found and, 1 to 2 months after surgery, the angiogram showed a renewed Adamkiewicz artery in another level [12]. However, the rate of this complication has been found to be from 0% to 0.75% [18, 19].

In 2011, Yazbeck et al. reported a case of irreversible paralysis after percutaneous vertebroplasty as cement leaked into the anterior spinal artery [24]. But no case of reversible anterior spinal artery syndrome after balloon kyphoplasty is to be found in the literature as yet.

There are many causes for an anterior spinal artery syndrome, for example, mikroangiopathia, dural-AV-fistula, vasculitis, or the injury/embolism of the Adamkiewicz artery described above. In this case, the pathomechanism is related to the boundary zones; circuit heparinization is indicated to inhibit platelet aggregation.

In conclusion, minimally invasive balloon kyphoplasty rarely has complications. To our knowledge, is the first reported case of a reversible anterior spinal artery syndrome after balloon kyphoplasty. But spine surgeons must be able to cope with this rare complication, using high-dose low-molecular heparin to reperfuse the Adamkiewicz artery and reducing the intra-abdominal pressure to avoid embolism. General prognosis of anterior cord syndrome is unfavorable [25], highlighting the relevance of this case report.
